# Editorial: Exploring circular RNAs and their applications within health and disease

**DOI:** 10.3389/fmolb.2023.1145738

**Published:** 2023-01-25

**Authors:** Matteo Becatti, Crismita Dmello, Binod Kumar

**Affiliations:** ^1^ Department of Experimental and Clinical Biomedical Sciences “Mario Serio”, University of Firenze, Firenze, Italy; ^2^ Department of Neurological Surgery, Northwestern Medicine Malnati Brain Tumor Institute of the Lurie Comprehensive Cancer Center, Feinberg School of Medicine, Northwestern University, Chicago, IL, United States; ^3^ Department of Antiviral Research, Institute of Advanced Virology, Thiruvananthapuram, Kerala, India

**Keywords:** circRNA, circular RNA, health, disease, therapeutics, splicing, ncRNA

The most recent advances in next-generation sequencing technologies have indicated that only 1.5% of the RNA transcribed from DNA is translated into protein. Circular RNAs (circRNA) are new non-coding RNA characterized by the presence of a covalent bond linking the 3′and 5′ends that have recently been identified in multiple cell types from diverse species. CircRNAs are generated *via* back-splicing or exon skipping of pre-mRNAs, a complex process regulated by intronic repeats and RNA-binding proteins (RBPs) that differs from canonical splicing (Ebbesen et al., 2016). CircRNAs have been described in various subcellular fractions (nucleus, cytoplasm, exosome), and are more stable than their linear ncRNA counterparts, because their structures are not affected by RNA exonuclease RNase R (Robinson and Port, 2022).

Although the functions of most circRNAs are not fully elucidated, emerging evidence demonstrate that dysregulated circRNAs play a key role in many biological processes as regulatory non-coding RNAs, by acting as microRNAs sponges, modulating splicing, transcription, chromatin modification, regulating RNA-binding proteins, as well as encoding proteins (Zhou et al., 2022). Their great stability, functional properties, and the presence in the circulation, make circRNAs not only a potential target but also therapeutic agents. This Research Topic collects some latest circRNAs diagnostic and therapeutic advances and their use in precision medicine and biomarker discovery and validation.

Accumulating evidence indicates that circRNAs are differentially expressed in cancer cells, and their dysfunctions are involved in tumorigenesis and metastasis; for this reason, they are promising cancer biomarkers. In this Research Topic, two review articles summarize the most up to date studies that analyze biogenesis, degradation and biological functions of circRNAs, as well as the potential clinical implications in renal cell carcinoma (Zhou et al.) and non-small cell lung cancer (Huang et al.). The identification of new biomarkers is essential for cancer drug development and therapeutic decision-making for individual patients, that translate the genomic information of individual tumors into a personalized therapeutic approaches. In this regard, Kong and collaborators investigated the tumor suppressor role of circLRRC7 in glioblastoma (Kong et al.). In this original article, through bioinformatic analysis followed by experimental validation, authors found that circLRRC7 was downregulated in glioblastoma patients when compared with non-tumor brain tissues, and proposed miR-1281 and PDXP as potential downstream genes of circLRRC7 in glioblastoma pathogenesis.

Bioinformatic tools are essential for data management in modern biology, life sciences, and medicine. In an interesting work, Cao and Li build up a novel circRNA prediction pipeline based on raw mass spectrometry data to explore the translation potential of circRNAs (Cao et al.). The tool contains a main program and several independent function modules that, starting with the data analysis of raw tandem mass spectrometry data, it can obtain hundreds of translatable circRNAs in humans and *Arabidopsis thaliana*.

Last but not least, Molibeli and collaborators reviewed the recent advances on the potential clinical application of exosomal circRNA (exo-circRNAs) (Molibeli et al.). Exosomes are membrane vesicles having a diameter of 30–150 nm, secreted into surrounding body fluids upon fusion of multivesicular bodies and the plasma membrane by different kinds of cells. They may contain proteins, circular RNAs (circRNAs), microRNAs, or DNA, and can modulate many intracellular pathways (Zhang et al., 2019). Exo-circRNAs play important role in cell proliferation, tumor progression and metabolism. Due to their presence in biological fluids, exo-circRNAs can be considered an important biomarker for the diagnosis, progression, and effective therapy for a range of diseases. In this review, Molibeli and co-workers summarise recent findings regarding exo-circRNAs, and highlight their potential applications and therapeutic targets in human diseases.

Together, the articles compiled in this Research Topic shed light on the current status of molecular diagnostic and therapeutic advances in the field of circRNAs.
